# Covalent Binding of Heparin to Functionalized PET Materials for Improved Haemocompatibility

**DOI:** 10.3390/ma8041526

**Published:** 2015-03-31

**Authors:** Metod Kolar, Miran Mozetič, Karin Stana-Kleinschek, Mirjam Fröhlich, Boris Turk, Alenka Vesel

**Affiliations:** 1 Jozef Stefan International Postgraduate School, Jamova 39, Ljubljana 1000, Slovenia; 2 Plasma Laboratory, Institute Jozef Stefan, Jamova 39, Ljubljana 1000, Slovenia; E-Mail: miran.mozetic@guest.arnes.si; 3 Faculty of Mechanical Engineering, University of Maribor, Smetanova 17, Maribor 2000, Slovenia; E-Mail: karin.stana@um.si; 4 Department of Biochemistry, Molecular and Structural Biology, Institute Jozef Stefan, Jamova 39, Ljubljana 1000, Slovenia; E-Mails: mirjam.frohlich@ijs.si (M.F.); boris.turk@ijs.si (B.T.); 5 Educell Ltd., Prevale 9, Trzin 1236, Slovenia

**Keywords:** poly(ethylene terephthalate), vascular graft, biocompatibility, heparin, plasma, functionalization, haemolysis, platelet adhesion, endothelization

## Abstract

The hemocompatibility of vascular grafts made from poly(ethylene terephthalate) (PET) is insufficient due to the rapid adhesion and activation of blood platelets that occur upon incubation with whole blood. PET polymer was treated with NH*_x_* radicals created by passing ammonia through gaseous plasma formed by a microwave discharge, which allowed for functionalization with amino groups. X-ray photoelectron spectroscopy characterization using derivatization with 4-chlorobenzaldehyde indicated that approximately 4% of the –NH_2_ groups were associated with the PET surface after treatment with the gaseous radicals. The functionalized polymers were coated with an ultra-thin layer of heparin and incubated with fresh blood. The free-hemoglobin technique, which is based on the haemolysis of erythrocytes, indicated improved hemocompatibility, which was confirmed by imaging the samples using confocal optical microscopy. A significant decrease in number of adhered platelets was observed on such samples. Proliferation of both human umbilical vein endothelial cells and human microvascular endothelial cells was enhanced on treated polymers, especially after a few hours of cell seeding. Thus, the technique represents a promising substitute for wet-chemical modification of PET materials prior to coating with heparin.

## 1. Introduction

Cardiovascular diseases represent the major cause of death in the modern world. Treating such diseases is often achieved by surgery, during which inefficient blood vessels are bypassed or replaced with synthetic ones. The surgery often leads to post-surgery complications, such as the formation of thrombi or improper growth of endothelial cells. These complications are due to the rather poor biocompatibility of currently used materials for vascular grafts. Currently, the grafts are made either from expanded polytetrafluoroethylene (ePTFE) or poly(ethylene terephthalate) (PET); the latter material performs better with respect to its mechanical properties. Vascular grafts made from knitted or woven PET fibers also allow for limited penetration of fluids and cells through the graft walls, which makes them preferred in current surgery practice. The major drawback of such artificial blood vessels, however, is poor hemocompatibility. Blood platelets recognize the foreign material and start activating on the surface. Adsorption of blood platelets is followed by the transformation of their shape, release of enzymes and factors and ignition of the protein-modification cascade [1]. A clot is formed and may eventually fill the vascular graft, requiring the surgery to be repeated. The problem is particularly severe for grafts of small diameters. The insufficient hemocompatibility of PET material is the driving force for research on modifications of the polymer surface properties using various approaches.

A suitable method for minimizing the risk of thrombosis is coating the PET material with anticoagulants [2,3]. The most popular anticoagulant is heparin, a glycosaminoglycan (GAG) that is rich in –SO_3_^−^ functional groups [4]. This GAG acts as an anticoagulant due to its binding to antithrombin (AT), the major inhibitor of coagulation proteases thrombin and Factor Xa, via a specific pentasaccharide sequence that contains a 3-*O*-sulfatesulfated glucosamine residue. This high-affinity pentasaccharide induces a conformational change in AT that accelerates the rate of factor Xa inhibition by at least two orders of magnitude but has very little to no effect on the rate of thrombin inhibition. The latter requires the formation of a ternary complex between thrombin AT and full-length heparin [5,6]. The polysaccharide is highly soluble in water, so it can be deposited onto a suitable substrate by dipping the sample into an aqueous solution. The problem, however, is the poor affinity of heparin for the polymer. The heparin film is quickly washed away from the polymer surface, so it resumes the insufficient hemocompatibility. The surface properties of the polymer substrate should therefore be modified to allow for good adhesion of the heparin film. Apart from wet-chemical methods such as aminolysis [7,8,9], gaseous plasma treatment is becoming increasingly popular [10,11,12,13,14,15,16,17].

Several authors have reported improved immobilization of heparin by treating the substrate with oxygen plasma [8,18,19,20,21,22]. Reactive oxygen species from plasma allow for rapid functionalization of the polymer surface with polar oxygen-rich functional groups, thus allowing for better wettability. The surface functional groups are negatively charged, similar to the groups on heparin. Therefore, a variety of additional treatments have been used in efforts to bind heparin firmly. Although rarely used, argon plasma has an effect similar to that of oxygen plasma treatment in improving wettability [23]. Heparin was bound using an interface of collagen.

Plasma created in ammonia has also attracted some attention as a method for the functionalization of polymers with amine groups [24,25,26]. The functionalized polymer can be deposited with albumin that is positively charged, followed by electrostatic stabilization of the negatively charged heparin [27]. Ammonia plasma has also been used for activation of diamond-like coatings on implants, followed by covalent grafting of heparin [28]. Additionally, ammonia has been mixed with argon to obtain the correct parameters for surface pre-treatment prior to deposition of heparin.

**Scheme 1:**
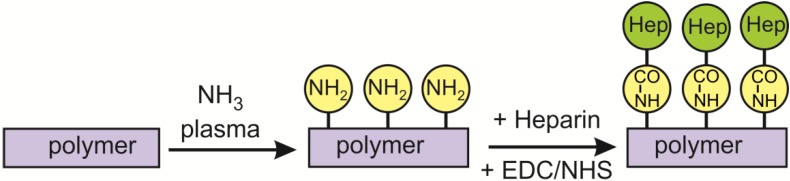
Multistep strategy for the molecular binding of heparin to ammonia plasma-treated poly(ethylene terephthalate) (PET) surface.

Treatment with gaseous plasma sustained in ammonia allows for the incorporation of nitrogen functional groups into the surface layer of polymers, but the groups may not be stable due to weak bombardment of the polymer surface upon plasma treatment. To avoid such unwanted effects, we performed functionalization using only neutral reactive particles found in ammonia plasma afterglow. After ammonia plasma treatment, heparin was covalently bound to the polymer surface according to Scheme 1. An amide bond is formed between the EDC/NHS (ethyl(dimethylaminopropyl) carbodiimide/N-hydroxysuccinimide) activated carboxyl group of heparin and primary amine on the surface [29].

## 2. Results and Discussion

### 2.1. Functionalization of the Polymer Surface with Amino Groups

Polymer samples were treated in the flowing afterglow of plasma created in several nitrogen-containing gases, including pure ammonia, a mixture of ammonia and argon, pure nitrogen and a mixture of nitrogen and hydrogen. X-ray photoelectron spectroscopy (XPS) spectra of treated samples revealed functionalization with nitrogen in all cases, but the concentration depended on the type of gas. Table 1 presents the composition of PET samples after a 30 s treatment in the flowing afterglow of plasma created at the same discharge parameters but with different gases. The concentration of nitrogen was much higher on samples treated with reactive particles from ammonia plasma (either with or without argon) than on other samples. This observation is explained by extensive formation of NH*_x_* radicals in ammonia plasma. In other plasma, such radicals are not likely to be formed. In particular, plasma created in the mixture of nitrogen and hydrogen causes dissociation of both types of molecules to parent atoms, but the atoms cannot associate to form NH radicals in the gas phase due to restrictions of classical physics—conservation of energy and momentum at the collision between the atoms. Both atoms associate to form molecules on the surfaces, but the association back to parent molecules is much more likely than the association of N and H atoms to form an NH radical [30]. Formation of NH_2_ radicals is marginal in the nitrogen-hydrogen mixture under plasma condition because the association of NH and H atom on the surface is forbidden in the gas phase due to the requirement of energy and momentum conservation.

**Table 1:** Composition of PET surface film (atomic%) after treatment with reactive particles from plasma of different gases.

Gas	C	N	O
untreated	74.7	-	25.3
NH_3_	64.7	10.6	24.7
NH_3_ + Ar	65.8	9.6	24.5
N_2_	61.2	2.9	35.8
N_2_ + H_2_	64.5	4.2	31.4

**Figure 1:**
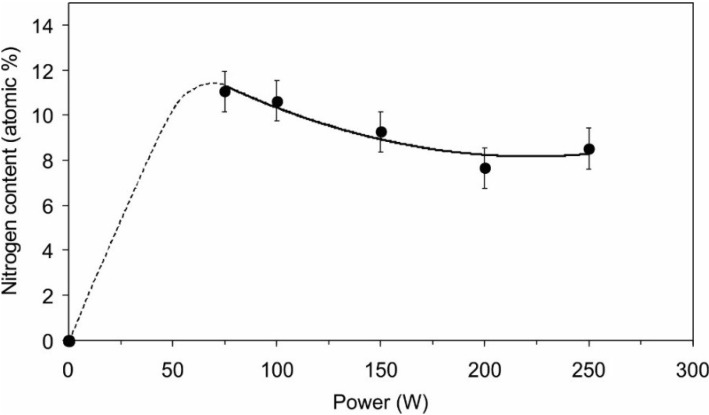
Concentration of nitrogen in PET surface film *versus* the power of the discharge generator for sustaining ammonia plasma.

The concentration of nitrogen in the surface film of the polymer, as determined by XPS, depends on the discharge power, as shown in Figure 1. Interestingly, the nitrogen concentration decreases with increasing treatment time. The concentration was determined only in the range of powers from 75 to 250 W due to the restrictions of the power supply. Plasma does not ignite at lower powers in our experimental configuration. The curve presented in Figure 1 is explained by the prevalence of different radicals at different discharge powers. At low powers, the plasma is limited to a small volume, and the electron energy and their density are relatively low. Therefore, the predominant dissociation event is e^−^ + NH_3_ → e^−^ + NH_2_ + H. Reactions such as e^−^ + NH_3_ → e^−^ + NH + 2H and e^−^ + NH_2_ → e^−^ + NH + H require larger electron energy and are thus less favorable at low discharge power. The NH_2_ radicals stick to the surface of polymer and remain bonded, forming the amino group. By increasing the discharge power, the dissociation of ammonia molecules becomes more efficient, and eventually the molecules would fully dissociate to N and H atoms at very high powers. Such power was not applied during testing so the concentration of nitrogen groups on the surface remained rather high even at the largest tested power of 250 W.

**Figure 2:**
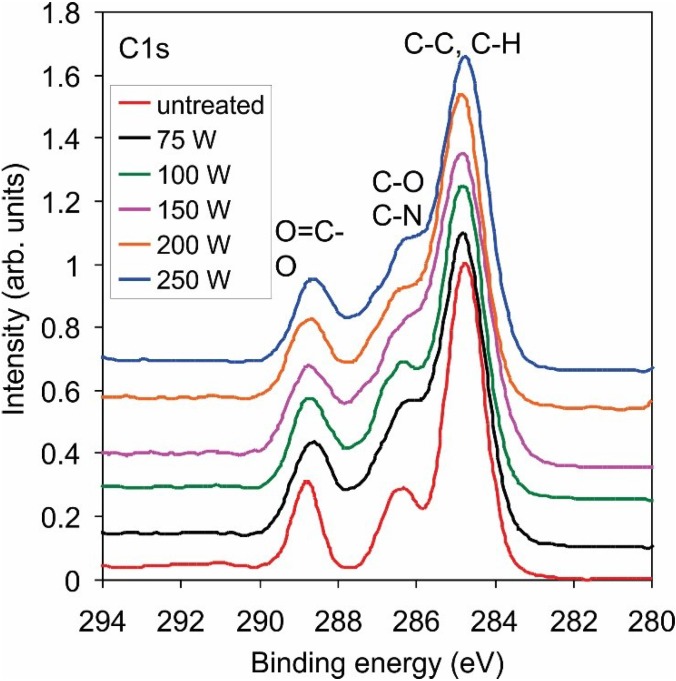
High-resolution C1s spectra for samples treated with reactive particles from ammonia plasma sustained at different powers.

**Figure 3:**
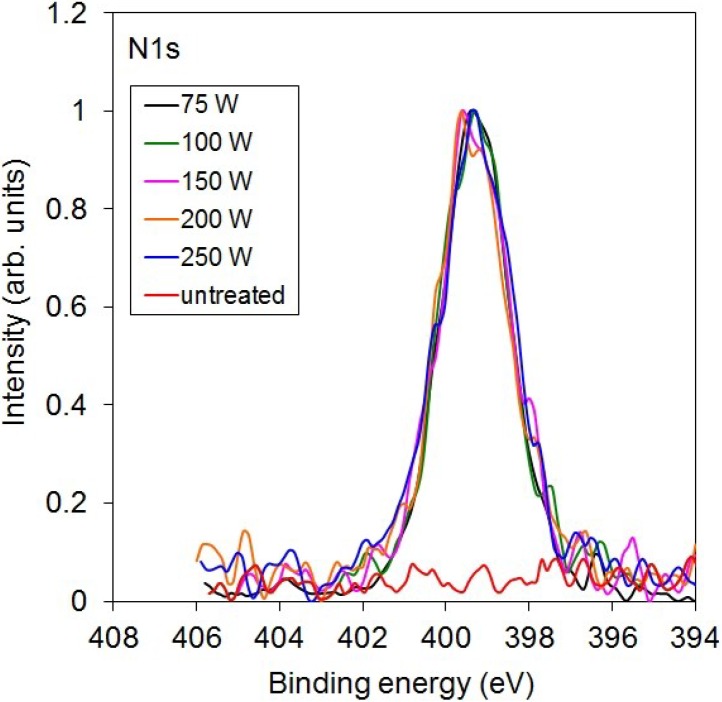
High-resolution N1s spectra for samples treated with reactive particles from ammonia plasma sustained at different powers.

**Figure 4:**
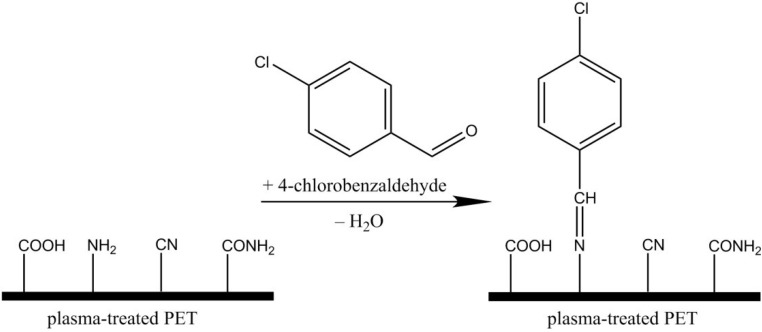
Schematic of 4-chlorobenzaldehyde binding to amino groups.

The XPS technique allows for determination of the surface composition but cannot distinguish between amino groups and other similar groups on the surface. Figure 2 presents the high-resolution C1s peak after treatment with radicals from ammonia plasma. Functionalization with nitrogen is clearly confirmed, but it is nearly impossible to determine the exact groups. Figure 3 shows the high-resolution N1s peak, and the curves are almost identical. To confirm the presence of amino functional group on the treated PET surface, we performed derivatization with 4-chlorobenzaldehyde [31,32]. Figure 4 presents the schematic of the technique, and Table 2 shows the concentration of elements after derivatization. The concentration of amino groups was determined from the XPS survey spectra of samples coated with 4-chlorobenzaldehyde using the following equation [32]:

(1) $$\left[{\text{NH}}_{2}\right]=\frac{\left[\text{Cl}\right]}{\left(\left[C\right]-7\left[\text{Cl}\right]\right)}$$

Table 3 shows the concentration of amino groups on the polymer surface after treatment with reactive particles from plasma that were created in ammonia at different powers as well in other nitrogen-containing gases.

**Table 2:** Composition of the PET surface film after binding 4-chlorobenzaldehyde (atomic%).

Power (W)	C	N	O	Cl
untreated	71.8		27.9	0.3
75	69.8	2.6	25.5	2.1
100	69.9	3.5	24.8	1.9
150	69.1	3.5	25.6	1.8
150	69.5	3.7	24.5	2.3
200	71.2	2.9	23.9	2.1
250	70.6	3.2	24.4	1.8

**Table 3:** Concentration of amino groups in PET surface film for different discharge powers using ammonia and different gases at 100 W power.

Power	NH_2_/%
untreated	0.4
75 W	3.8
100 W	3.4
150 W	3.7
200 W	3.7
250 W	3.1
NH_3_ + Ar	4.3
N_2_	0.8
N_2_ + H_2_	1.3

Some chlorine was found even on the surface of untreated samples, so the technique has limited reliability. However, the results summarized in Table 3 clearly demonstrate the superiority of ammonia compared with the other gases. The concentration of amino groups is almost 4% in all samples treated by reactive particles originating from ammonia plasma.

**Figure 5:**
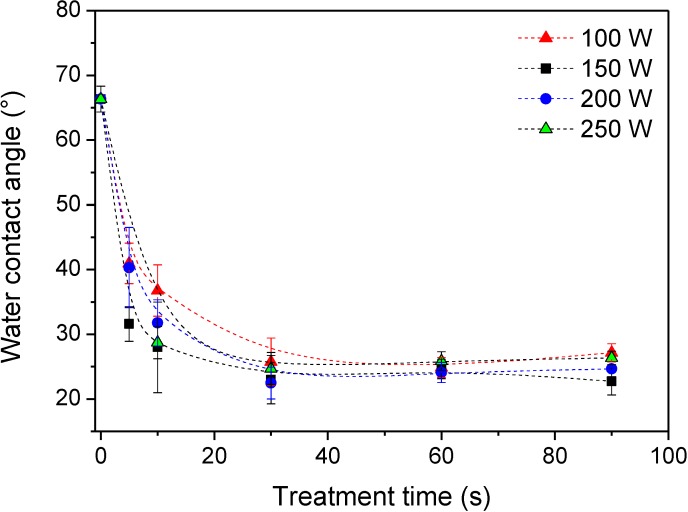
Water-drop contact angles for samples treated with reactive particles from ammonia plasma.

Treatment of PET samples with reactive particles from ammonia plasma also allowed for improved wettability of the polymer. Figure 5 shows the contact angle of a water drop after treatment with different discharge powers and periods. The polymer is originally moderately hydrophobic, and the contact angle is close to 70°. Even a short treatment time causes a decrease of the contact angle. After 10 s of plasma treatment the angle is approximately 30°, more for low discharge powers and less for high powers. After half a minute, any dependence of the contact angle becomes marginal, and the contact angle assumes the value of approximately 25°. The improved wettability is favorable because heparin was deposited from a water solution.

### 2.2. Covalent Binding of Heparin

**Figure 6:**
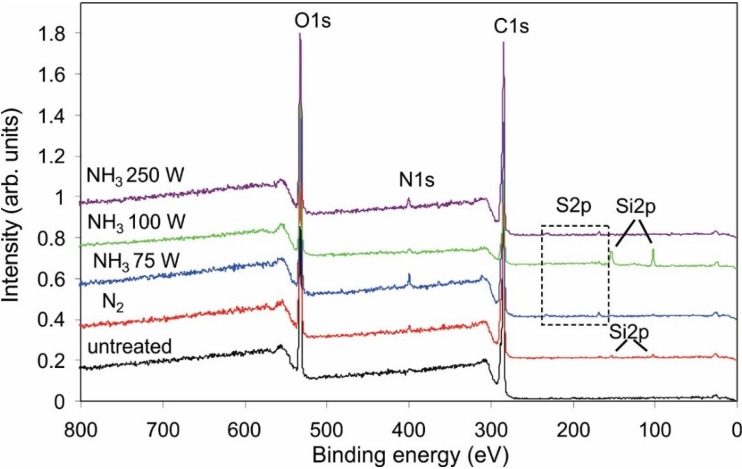
X-ray photoelectron spectroscopy (XPS) survey spectra for polymers treated under different conditions.

**Figure 7:**
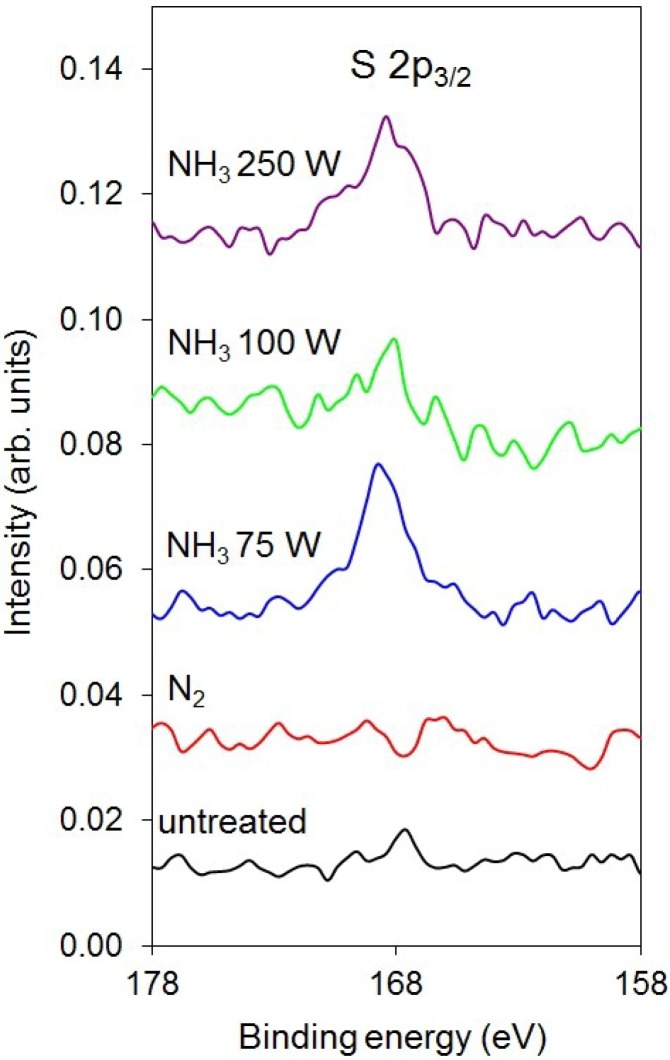
Enlarged XPS survey spectra in the energy range where the S 2p_3/2_ peak appears.

Figure 6 presents the XPS survey spectra after deposition of heparin on polymer samples that were previously treated with reactive particles from plasma created in different nitrogen-containing gases. Apart from the existence of nitrogen on the surface of PET samples that were pre-treated with reactive particles from ammonia plasma, no significant differences are observed. The composition of the surface film deduced from the survey spectra is presented in Table 4. The concentration of nitrogen is rather small in all samples after the deposition of heparin, indicating either the loss of nitrogen groups on the surface upon dipping into the heparin solution or screening due to the heparin coating. The small concentration of sulfur, which is typical for heparin, could be an artifact of the measurement. To confirm that sulfur actually exists on the surface of some samples, we present enlarged XPS spectra in Figure 7. In this figure, only the part of the spectra between 158 and 178 eV is shown. The peaks observed for the cases of pre-treatment with reactive particles from ammonia plasma are well-distinguished from the background and appear at the binding energy where the S 2p_3/2_ should appear. Figure 7 presents firm evidence of sulfur on selected samples.

**Figure 8:**
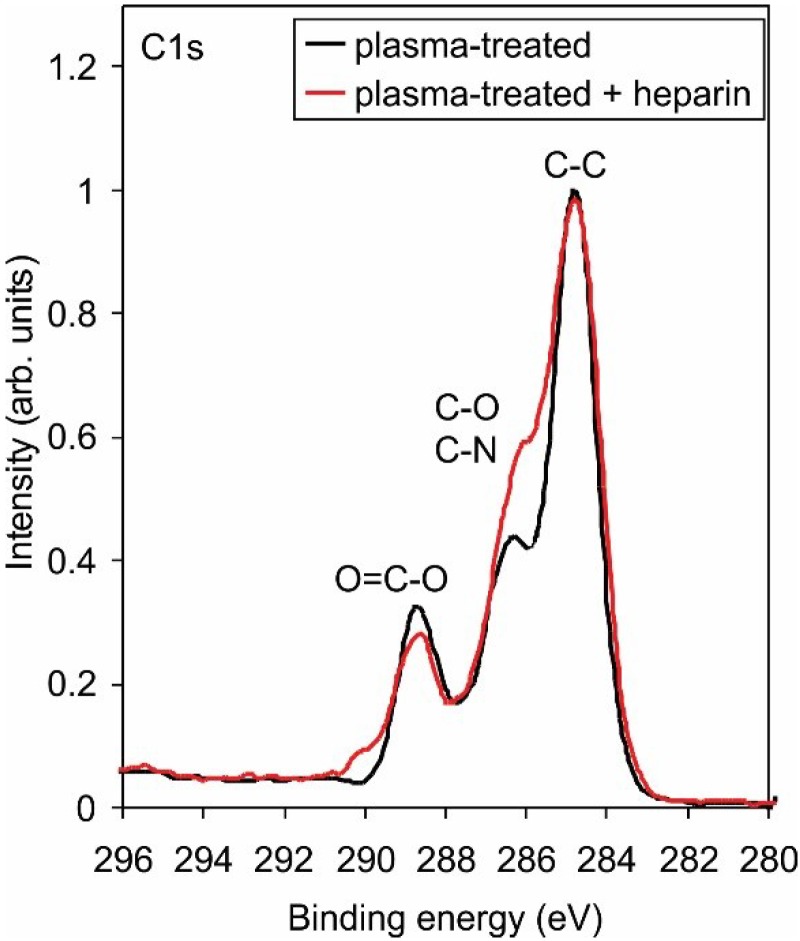
High-resolution C1s spectra for samples with amino groups before and after depositing heparin.

The high-resolution C1s peak for a sample treated only with reactive particles from ammonia plasma and a sample treated in same way but after dipping into heparin solution is shown in Figure 8. The peaks are normalized to the main peak corresponding to the C–C bonds. Distinguished differences, especially at the binding energy corresponding to the C–O group, are observed. The result is explained by considering the heparin structure. This polysaccharide contains numerous C–O bonds and is therefore the reason for the differences between the curves presented in Figure 8.

**Table 4:** Surface composition of polymers after incubation with heparin (atomic%).

Sample	C	N	O	S
untreated	75.1	-	24.9	-
N_2_-100 W	71.3	1.5	27.2	-
NH_3_-75 W	70.4	2.9	26.0	0.7
NH_3_-100 W	63.3	2.2	33.8	0.7
NH_3_-250 W	70.9	2.0	26.5	0.6

### 2.3. Biological Response

**Figure 9:**
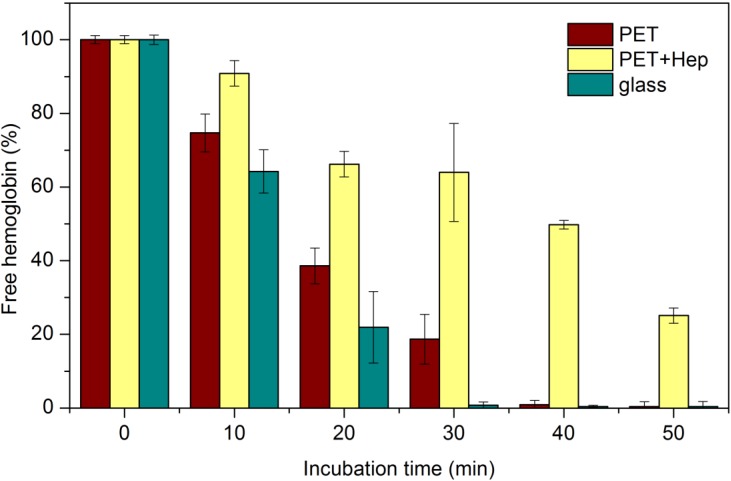
Concentration of free haemoglobin after different incubation periods of samples with whole blood.

The samples were incubated with fresh blood, and their hemocompatibility was tested by the free-hemoglobin method [33]. Figure 9 presents the time dependence of the free hemoglobin concentration after the incubation. The value at time zero was set to 100%. Results are presented as the mean ± standard deviation calculated on the basis of five parallel measurements. The concentration of free hemoglobin decreases with time after incubation for both samples but decreases more rapidly for the untreated PET. For these untreated samples, the concentration decreased to approximately 75% 10 min after incubation, thus confirming the insufficient hemocompatibility of untreated PET materials. The decrease is steep, and after 40 min the concentration of free hemoglobin in the liquid medium dropped below the detection limit of the method.

The samples pre-treated with the reactive gaseous particles from ammonia plasma and coated with heparin show different behavior. Thirty minutes after incubation, the concentration of free hemoglobin resulting from the haemolysis of erythrocytes is still well above 50% of the original value. The ratio of hemoglobin concentration between treated and untreated polymer samples increases with time after the incubation. After 20 min, the concentration is approximately twice as high as in the untreated samples, and it increases to 4 times greater after 30 min; for longer times, the ratio approaches infinity because the concentration for the case of untreated samples approaches zero. These results illustrate the favorable hemocompatibility of materials with functionalized polymer surfaces with amino groups treated by ammonia plasma flowing afterglow followed by covalent binding of a thin heparin layer onto the groups, which act as anchor sites for the heparin molecules.

**Figure 10:**
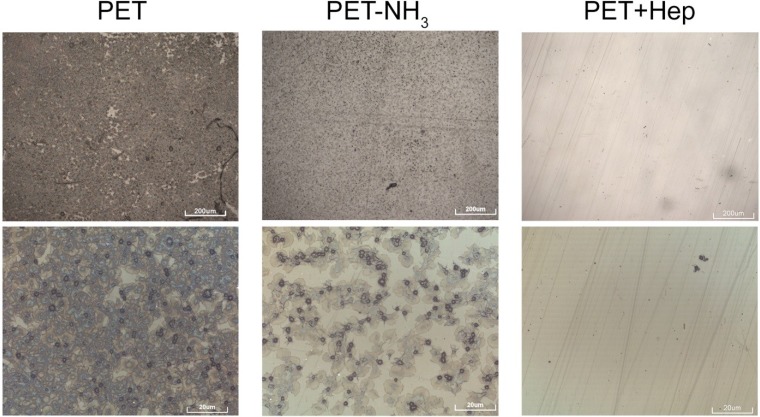
Optical images of samples after incubating with whole blood.

As haemolysis is not considered to be a good marker for haemocompatibility determination, additional investigation of platelet adhesion was performed. For the *in vitro* studies, untreated (PET), ammonia plasma treated (PET-NH_3_) and ammonia plasma treated with immobilised heparin (PET + Hep) were incubated with fresh whole citrated blood as described below. Numerous samples were prepared to check the repeatability of results and the most representative images are shown in Figure 10. The surface is covered with platelets in different morphological forms; according to the literature [34], the round shaped ones are in a non-activated form, while those that appear in spread dendritic or fully spread form are activated. A surface of a sample PET is almost completely covered with platelets. A higher magnification image shows that the adhered platelets are in fully spared form, thus concluding that a major activation of platelets has taken place. On sample PET-NH_3_ lesser number of adhered platelets can be observed compared to the untreated PET. In addition the platelets are not fully activated, since other morphological forms can be seen, such as spread dendritic and spreading form. Contrary to previous samples significantly lower amount of adhered platelets can be observed on the sample with immobilised heparin (PET + Hep). The adhered platelets are mostly in round or dendritic forms. The results summarized in Figure 10 therefore indicate a significant decrease in number of adhered platelets on surfaces of PET with immobilised heparin compared to untreated PET surfaces.

**Figure 11:**
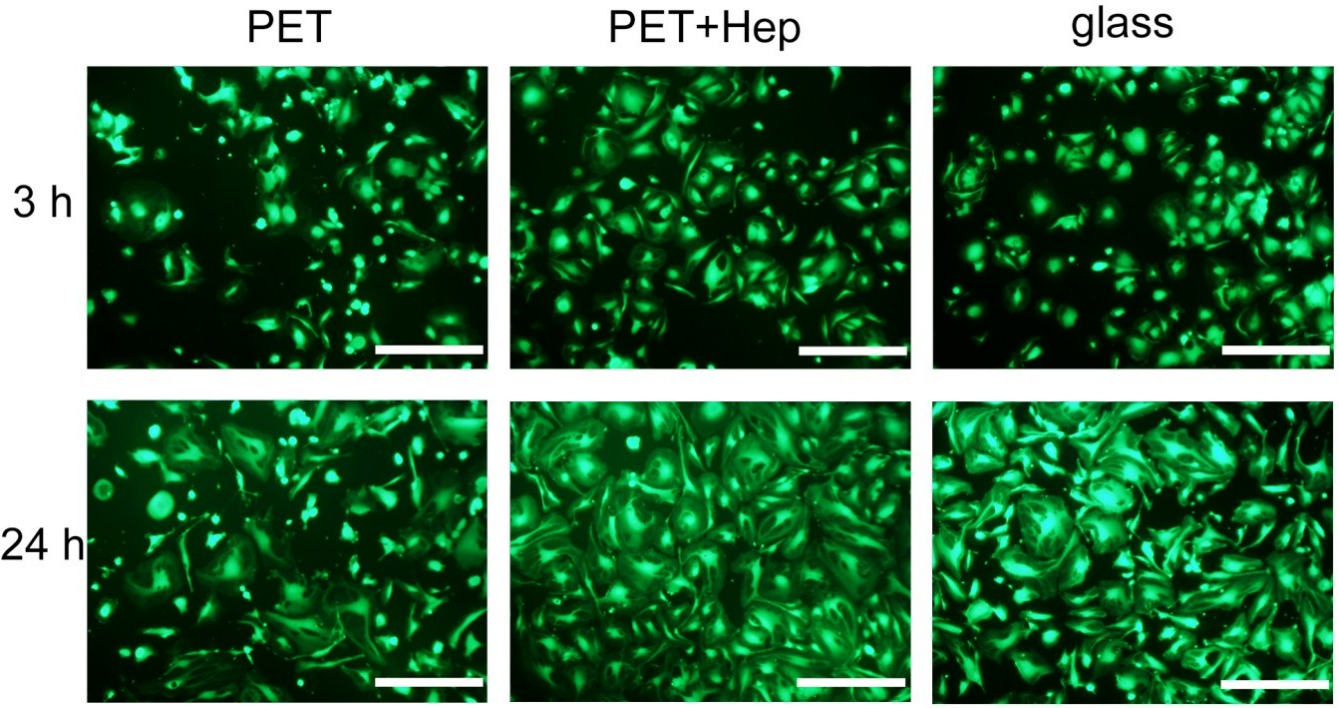
Optical images of samples 3 and 24 h after seeding HUVECs. Scale bar represents 500 μm.

**Figure 12:**
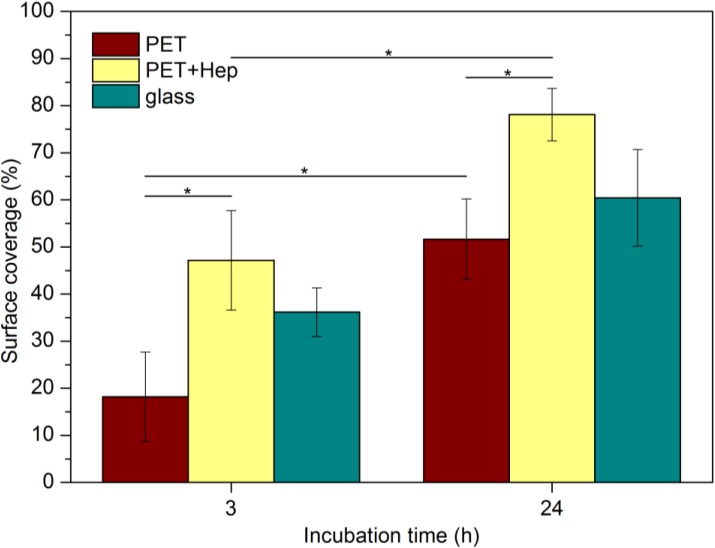
Surface coverage with HUVECs on different substrates (* *P* < 0.05).

Only endothelial cells are considered to determine the best suitable blood-contacting material; therefore, the rapid endothelisation of cardiovascular implants made from polymers is preferred. To study the ability of heparin-coated PET to support the attachment and growth of endothelial cells, we performed endothelisation experiments with human umbilical vein endothelial cells (HUVECs) and human microvascular endothelial cells (HMVECs). Cells were seeded onto both untreated and treated polymers as well as glass, a commonly used surface for *in vitro* experiments that enables good adhesion and proliferation of endothelial cells at appropriate conditions. Figure 11 shows typical optical microscopy images of the samples at 3 and 24 h after HUVEC seeding. Cells were stained with the LIVE/DEAD^®^ Viability kit (Life Technologies, Grand Island, NY, USA) to identify live and dead cells. No, or minimal numbers, of dead cells were found under all tested conditions. However, the differences in the areas covered by live cells between the different surfaces are clearly visible. Three hours after seeding, the polymer samples treated with reactive particles from ammonia plasma and coated with heparin exhibit much better coverage compared with untreated PET. Cell morphology is also clearly different on untreated PET in comparison to heparin-coated PET. While cells on heparin-coated PET attach very well and acquire typical endothelial cell-cell contacts, many cells are rounded when cultured on PET, indicating less firm attachment. The optical images presented in Figure 11 allow for determination of the surface coverage, and the results are shown in Figure 12. Large differences are evident between untreated and treated polymers; the surface coverage after 3 h is approximately 2.5-fold larger for treated than untreated samples. The coverage is even larger than in the case of the glass substrate, but the difference is close to the standard deviation, as indicated with error bars in this figure. The difference at 24 h after cell seeding is not remarkable because cell proliferation is progressing in all cases. However, PET-HEP remains a superior substrate to PET. The results summarized in Figure 12 therefore indicate that the initial cell adhesion and subsequent proliferation is much better for heparin-coated samples than for untreated polymer and glass.

**Figure 13:**
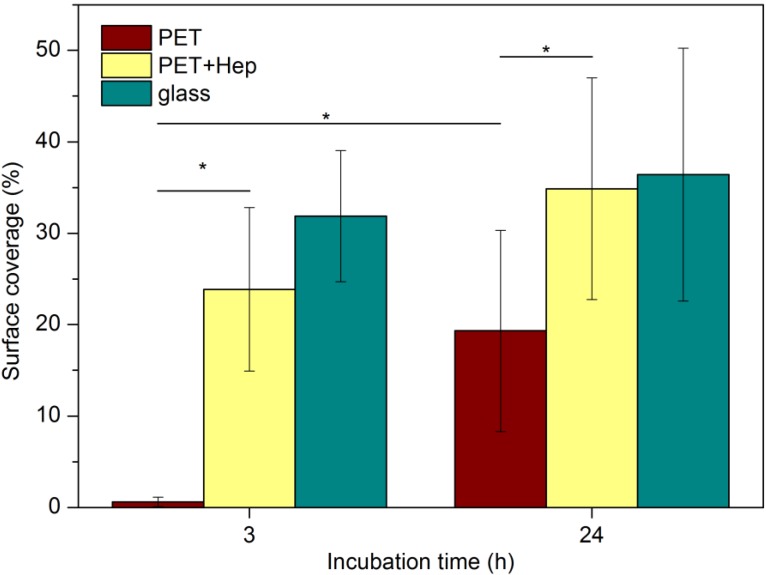
Surface coverage with HMVECs on different substrates (* *P* < 0.05).

Similar results were obtained with HMVECs and are shown in Figure 13. For HMVECs, the differences in adhesion are dramatic at three hours after cell seeding. While the surface coverage is estimated to be approximately 23% and 32% for treated polymer and glass, respectively, the coverage remains below 5% for untreated polymer. This finding indicates poor adhesion of the HMVECs on untreated polymer. The results obtained for treated polymer are similar to those for glass when considering the statistical error. The differences are less pronounced 24 h after seeding, but the coverage is still almost twice the size for treated polymer compared with untreated.

## 3. Experimental Section

### 3.1. Plasma Treatment

**Figure 14:**
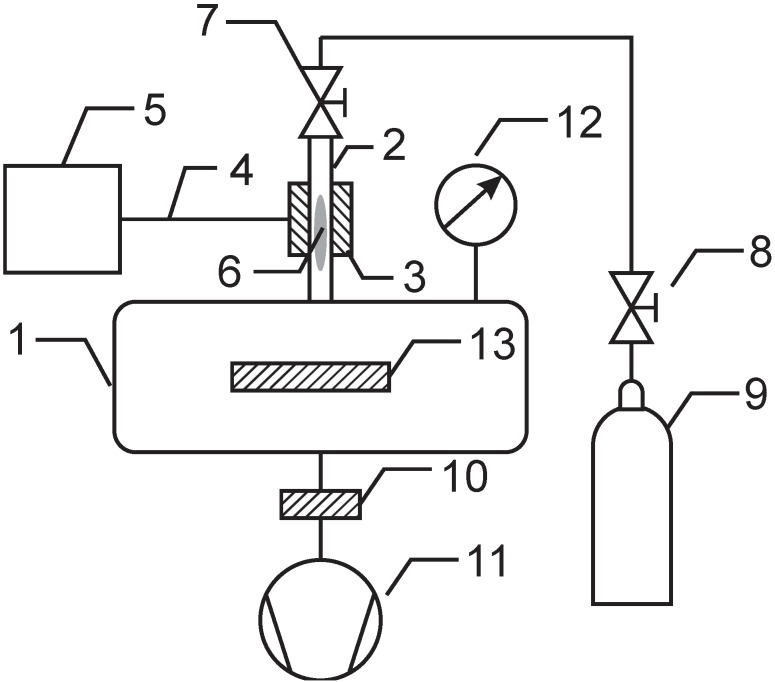
Schematic of the experimental setup. 1: processing chamber, 2: quartz tube, 3: microwave cavity, 4: coaxial cable, 5: power supply, 6: plasma, 7: flowmeter, 8: high-pressure valve, 9: ammonia flask, 10: catalyzer, 11: vacuum pump, 12: vacuum meter, 13: polymer sample.

Polymer samples (PET from Goodfellow Cambridge Ltd., Huntingdon, UK) were functionalized in an experimental system that is presented schematically in Figure 14. The vacuum system was pumped with a two-stage rotary pump of nominal pumping speed 60 m^3^/h and ultimate pressure of approximately 0.1 Pa. The pump was connected to a processing chamber via a catalyzer. The catalyzer contained copper and nickel meshes and allowed for recombination of radicals to stable molecules before entering the pump. The processing chamber was made from Pyrex glass. The sample was mounted into the center of the processing chamber below the junction with a quartz tube. There was an absolute pressure meter connected to the processing chamber. The pressure in the processing chamber was set to 50 Pa. A microwave cavity of approximately 5 cm in length was mounted onto the quartz tube of inner diameter 6 mm, as shown in Figure 14, and connected to the power supply via a coaxial cable. The microwave power was varied from 75 to 250 W. The length of the quartz tube between the microwave cavity and exhaust to the processing chamber was 10 cm. Gas was leaked into the quartz tube through an adjustable flowmeter. Different nitrogen containing gases were used (nitrogen and its mixture with hydrogen, ammonia and its mixture with argon). Due to continuous pumping on one side and gas leakage on the other one, there was a pressure gradient along the quartz tube. The gradient allowed for a high speed of gas through the tube. Rather homogeneous plasma was created in the quartz tube within the microwave cavity, and the luminous gas expanded approximately 1 cm away from the cavity toward the exhaust of the quartz tube. Such a configuration allowed for the rapid transport of gaseous species created in plasma to the processing chamber.

Within the cavity, the density of charged particles was high enough to sustain gaseous plasma. Once the gas drifted from the region toward the processing chamber, the density of charged particles decreased along the tube due to neutralization because practically no electromagnetic field was present outside the cavity. The neutralization was very efficient; therefore, only neutral reactive particles reached the surface of the polymer. In the case of ammonia, the particles were NH*_x_* and H radicals. The ammonia flask was replaced occasionally with flasks containing other gases, such as NH_3_ + Ar, pure nitrogen and N_2_ + H_2_. In such cases, the production of NH*_x_* radicals in plasma was not as extensive as in the case of ammonia and was absent in the case of pure nitrogen.

### 3.2. Surface Characterization

The composition and structure of the surface layer on the polymer samples was determined by high-resolution XPS. All analyses were performed soon after treatment of samples to minimize the influence of any ageing effects. We used a TFA (Thin Film Analysis) XPS instrument from Physical Electronics Inc. (Chanhassen, MN, USA). The base pressure in the XPS analysis chamber was approximately 6 × 10^−8^ Pa. The samples were excited with X-rays over a 400-μm spot area with monochromatic Al K_α1,2_ radiation at 1486.6 eV. The photoelectrons were detected with a hemispherical analyzer positioned at an angle of 45° with respect to the normal sample surface. The energy resolution was approximately 0.5 eV. Survey-scan spectra were acquired at a pass energy of 187.85 eV, while individual high-resolution spectra were taken at a pass energy of 23.5 eV with a 0.1 eV energy step for C1s. Because the samples were insulators, an additional electron gun was used to allow for surface neutralization during the measurements. All spectra were referenced to the main C1s peak of the carbon atoms, which was assigned a value of 284.8 eV. The spectra were analyzed using MultiPak v8.1c software (Ulvac-Phi Inc., Kanagawa, Japan, 2006) from Physical Electronics, which was supplied with the spectrometer.

### 3.3. Heparin Immobilisation

Covalent binding of heparin (sodium salt, MW (molecular weight) = 15 ± 2 kDa, Merck Millipore, Darmstadt, Germany) to the surface of polymers that had been functionalized with amino groups was performed by dipping the samples into a suitable water solution (see Scheme 1). The solution contained 1% heparin in 50 mM MES buffer. To facilitate covalent bonds between amino groups on the polymer surface and carboxyl groups of heparin, the crosslinking agent *N*-(3-dimethylaminopropyl)-*N′*-ethylcarbodiimide hydrochloride (EDC) was added at a concentration of 6 mM. The binding was stabilized using *N*-hydroxylsulphosuccinimide (NHS) at a concentration of 3.5 mM. The samples were left in the solution for at least 4 h. Next, the samples were thoroughly rinsed, first with an aqueous solution of 3 M NaCl and then with Milli-Q water (Merck Millipore, Darmstadt, Germany). Finally, samples were dried at ambient conditions. All chemical reagents were supplied by Sigma Aldrich Co. (Taufkirchen, Germany), unless otherwise specified.

### 3.4. Blood Incubation and Endothelisation

Incubation with fresh whole blood from healthy donors was performed as follows. Blood was withdrawn via vein puncture and used fresh without cooling or the addition of an antithrombotic agent. Incubation of polymer samples was performed in 25 mL glass containers in a warm water bath at 37 °C. Exactly 0.1 mL of fresh blood was placed on each sample. After defined times (10, 20, 30, 40 and 50 min), clotting was blocked by adding a defined amount of phosphate buffer to the beaker. Red blood cells that were not entrapped within the formed blood clot were hemolyzed, and free hemoglobin was dispersed into the liquid. The concentration of free hemoglobin was determined by measuring the absorbance at 540 nm (Cary 60 UV-Vis, Agilent, Santa Clara, CA, USA). Five parallel samples were analyzed for the statistical evaluation of measurement results.

Platelet adhesion experiments were performed using whole blood, collected from a healthy human volunteer. Blood was withdrawn with 21-gauge needle into evacuated tube containing 3.2% sodium citrate as anticoagulant. Samples were incubated with 1ml of fresh whole human blood in 24-well cell culture plate with shaking at 250 RPM for 1 h at 37 °C. After incubation the samples were rinsed several times with phosphate buffer saline (1× PBS) in order to remove blood residues and treated with 2.5% glutaraldehyde. After fixation the samples were rinsed with deionized water and left to air dry. All experiments were performed in triplicates and were repeated at least twice. The adhesion of platelets on samples was observed by confocal microscopy. A high resolution Axio CSM 700 (Carl Zeiss, Jena, Germany) confocal light microscope was used.

Endothelization was studied for two types of primary cells that were obtained from Life Technologies Corporation (New York, NY, USA): HUVECs and HMVECs. The samples were washed with sterile phosphate-buffered saline (PBS) and then placed in a 24-well tissue culture plate. Cells were seeded in wells at a cell density 5000 cells/cm^2^; a cell suspension in medium 131 supplemented with MVGS (microvascular growth supplement) and 0.05 mg/mL gentamicin (all from GIBCO^®^ from Life Technologies Co.) was added to each well, and the cultured in a humidified atmosphere of 5% CO_2_/95% air at 37 °C. After a certain incubation period (3 and 24 h), the samples were removed from the well and stained with the LIVE/DEAD^®^ Viability kit (Live: calcein AM, green signal; dead: ethidium homodimer-1, red signal). Cell morphology was observed with an inverted fluorescent microscope (IX71, Olympus, Tokyo, Japan). The covered area was quantified with computer software (ImageJ). Approximately 6–13 images were analyzed per each of the two parallel of samples for statistical evaluation. Statistical comparisons were performed using one-way analysis of variance (ANOVA) with the Tukey HSD post hoc analysis, where *P* < 0.05 was accepted as significant. Significant differences are indicated with an asterisk (*) in Figure 12 and Figure 13.

## 4. Conclusions

The application of flowing afterglow instead of glowing plasma allowed for the functionalization of PET materials with amino groups. An appropriate discharge power was used for partial dissociation of ammonia molecules. The NH_2_ and NH radicals do not associate extensively to form NH_3_ molecules on the way from plasma to the processing chamber, where they are allowed to interact with the polymer materials. Almost half of the nitrogen-containing functional groups on the polymer surface were amino groups, as determined by XPS using derivatization with 4-chlorobenzaldehyde. The density of the anchor sites on the polymer surface was high enough to allow for uniform covalent binding of heparin. The free hemoglobin method was used to determine the hemocompatibility of such samples, and the results clearly demonstrate almost optimal properties of materials prepared with this method. In addition to longer coagulation time, a significant decrease in number of adhered platelets was observed on such samples. The treated samples were endothelized much faster than the untreated samples. Therefore, the technique may be suitable for surface finishing of vascular grafts used in current medical practice.
